# Malocclusion in deciduous dentition: a cross-sectional study in a Portuguese preschool population

**DOI:** 10.1007/s40368-024-00935-1

**Published:** 2024-08-29

**Authors:** C. Esperancinha, S. Mendes, M. Bernardo

**Affiliations:** Faculdade de Medicina Dentária, Universidade de Lisboa, Unidade de Investigação Em Ciências Orais E Biomédicas (UICOB), 1600-277 Lisbon, Portugal

**Keywords:** Malocclusion, Prevalence, Deciduous dentition, Dental occlusion, Preschool child

## Abstract

**Purpose:**

To determine the prevalence of malocclusion in Portuguese preschool children, to characterise occlusion in the deciduous dentition, and to study the relationship between malocclusion, sex, and age.

**Methods:**

This cross-sectional study included 3–6 years old children, from 25 randomly selected kindergartens from the Lisbon district. Data were collected through an oral examination. Malocclusion was defined as the presence of any type of occlusion anomaly (anterior crossbite, edge-to-edge, increased overjet, open bite, deep overbite, posterior crossbite, scissor bite), spacing anomaly (crowding), or dentition anomaly (supernumerary teeth, agenesis, malformations). Canine class, terminal plane of the deciduous second molars and type of Baume arch were also recorded. Statistical analysis included descriptive and chi-squared test (*α* = 0.05).

**Results:**

The sample included 426 children with a global prevalence of malocclusion of 60.6%. Occlusion anomalies were the most prevalent (59.2%), the most frequent being deep overbite (27%), increased overjet (22.1%) and posterior crossbite (14.1%). Crowding had a prevalence of 1.6% and agenesis of 1.4%. Class I canine (57.3%), straight second molar terminal plane (60.1%) and type I Baume arch (53.3%) were the most common. The mean overjet was 2.6 mm (sd = 2.1) and the mean overbite was 2.2 mm (sd = 2.1). Age was associated with the presence of occlusion anomalies (*p* = 0.03), and increased overjet prevalence was found to be higher in girls (*p* = 0.03).

**Conclusions:**

The prevalence of malocclusion was high, with occlusion anomalies being the most prevalent. It is important to identify modifiable risk factors associated with malocclusion to prevent this condition in both the deciduous and permanent dentitions.

## Introduction

Malocclusion can have aesthetic consequences on the teeth and face, and functional consequences in chewing, swallowing, phonetics, posture and temporomandibular joint (Simões 2003; Tesch et al. 2004). Because of the significant impact on the quality of life of affected individuals, of the costs associated with its treatment and of its high prevalence all over the world, malocclusion is considered a public health problem (Dimberg et al. 2015a; Freire et al. 2022).

Malocclusion originates from the complex interaction between numerous stimuli during the formation and development of the orofacial complex in childhood and adolescence. These stimuli can be related to genetic, behavioural, and environmental factors (van der Linden 1966; Planas 2013). Harmful habits and other conditions in early childhood, such as prolonged non-nutritive sucking habits, other deleterious habits, premature tooth loss, oral and respiratory infections, among others, are considered risk factors for the development of malocclusion (Paolantonio et al. 2019; de Deus et al. 2020; Sight and Jawdekar 2024).

Malocclusion in the permanent dentition is related to its development in the deciduous dentition (Bishara et al. 1995; Onyeaso and Sote 2002; Peres et al. 2015), a reason why early diagnosis of this condition, including its associated factors, is paramount. In the deciduous dentition, the existence of type I Baume’s dental arches, primate spaces and either straight or mesial step terminal plane of the deciduous second molars, contribute to the establishment of a normal occlusion in the mixed and permanent dentitions (Cândido et al. 2010).

One of the major challenges in carrying out epidemiological studies of malocclusion is the absence of generally accepted diagnostic criteria/classification. Although there have been some attempts in the past to create standardized criteria (Björk et al. 1964; FDI 1973; Baume and Maréchaux 1974), a great diversity of criteria is still found in the different studies available in the literature. Various malocclusion indices have been proposed since the end of the nineteenth century, however, most were developed for the permanent dentition (Moimaz et al. 2021). Summers' Occlusal Index was designed for the deciduous dentition but does not consider skeletal or functional problems and is difficult to apply (Grippaudo et al. 2014).

The criteria of Björk et al. (1964) were developed specifically to be used in children and for epidemiological purposes. The registration of malocclusion is divided into three parts, namely, anomalies in the dentition (tooth anomalies, abnormal eruption, and misaligned teeth); occlusal anomalies (deviations in the positional relationship between the upper and lower arches); and deviations in space conditions (spacing or crowding). Despite their age, the Björk et al. criteria are still widely used (Hassan and Rahimah 2007).

Malocclusion in the deciduous dentition has a very varied prevalence. In the literature, prevalence rates vary widely, with a minimum of 31.6% in a study carried out in Iran (Jahanimoghadam et al. 2021) and a maximum of 83.9% in a study carried out in China (Zhou et al. 2017). In European countries, the prevalence of malocclusion in the deciduous dentition is also high, with figures of 74.7% in Germany (Grabowsky et al. 2007), 71% in Sweden (Dimberg et al. 2015b), 50% in Italy (Grippaudo et al. 2020), 46.2% in Spain (Amr-Rey et al. 2022) and 78.8% in Portugal (Furtado 2008). Nonetheless, the use of different criteria makes the comparison of the results challenging.

The epidemiological study of malocclusion in the deciduous dentition is important in planning the most appropriate preventive measures and implementing early orthodontic treatment. These efforts are essential to promote an increase in the percentage of the population with normal occlusion, reducing the proportion of moderate or severe forms of malocclusion to acceptable levels. However, epidemiological studies of malocclusion in the deciduous dentition are scarce and use mostly non representative samples; therefore, the objectives of this study were: (1) To determine the prevalence of malocclusion in a preschool population; (2) To analyse the characteristics of occlusion in the deciduous dentition; (3) To study the association of malocclusion with sex and age.

## Materials and Methods

To achieve the proposed objectives, a cross-sectional study was carried out with the approval of the Ethics Committee of the Faculty of Dental Medicine of the University of Lisbon (#20,190,250).

A pilot study was previously made on a non-probabilistic sample of around 90 children (Pimenta et al. 2023), which served as training and calibration for the observer and to rehearse the fieldwork.

The study's target population consisted of children aged between 3 and 6 years old, attending kindergartens in the Lisbon District (Portugal). A minimum sample size of 382 children was calculated considering the size of the target population (*N* = 62,390), the estimated prevalence of malocclusion (50%), a 95% confidence interval and a 5% margin of error. Sampling was performed using a two-stage random method, resulting in the selection of 25 kindergarten classrooms from the 16 municipalities of the Lisbon District. The choice of schools was proportionally stratified by type of kindergarten (public, private or Private Social Solidarity Institution—PSSI)) and by municipality. Due to their lower population, the municipalities of Azambuja, Arruda dos Vinhos, Cadaval, Sobral de Monte Agraço, Lourinhã, and Alenquer, were grouped together.

All the participating institutions gave permission for the study to be carried-out and for data to be collected on their premises. All the children in the selected classrooms with consent of their parents or legal guardians, who assented to take part in the study, and who had exclusive deciduous dentition were included. Children who had undergone or were undergoing orthodontic treatment were excluded.

Data collection included oral examination and the recording of the sex and age of the participating children, and took place in 2019 and 2022, as data collection had to be interrupted in 2020 and 2021 due to the COVID-19 pandemic.

The oral examination was made using natural and artificial light, a periodontal probe (CPI), an intraoral mirror and a millimetre ruler. The team consisted of two members, a previously calibrated examiner, and a recorder, both using the necessary equipment and materials to prevent cross-infection. The oral examination included the assessment of occlusal characteristics and diagnosis of malocclusion.

The criteria used to study malocclusion in the deciduous dentition were based on the criteria of Bjork et al. (1964), Zhou et al. (2016) and Grippaudo et al. (2014). Three main groups of anomalies were considered (occlusion, spacing, and dentition). Occlusion anomalies included sagittal, vertical, or transversal problems. In the sagittal plane, increased overjet, edge-to-edge, and anterior crossbite were assessed. In the vertical plane, deep overbite and open bite were assessed. Posterior crossbite and scissor bite were assessed in the transversal plane. Spacing anomalies included crowding, and finally, dentition anomalies included the presence of supernumerary teeth, agenesis, and malformed teeth. To characterize occlusion in the deciduous dentition, the following occlusal parameters were assessed and/or measured: overbite, overjet, canine class (I, II or III), terminal plane of the deciduous second molars (straight, distal, or mesial), and Baume arch type (I or II). Table 1 shows the malocclusion criteria used in this study.

**Table 1:** Criteria for malocclusion in the deciduous dentition

Occlusion anomalies	Sagittal anomalies	Overjet < 0 mm	Anterior crossbite
		Overjet = 0 mm	Edge-to-edge
		Overjet > 3 mm	Increased overjet
	Vertical anomalies	Overbite < 0 mm	Open bite
		Overbite > 3 mm	Deep overbite
	Transversal anomalies	Buccal cusps of one or more upper posterior teeth occlude in the central groove of the lower posterior teeth	Posterior crossbite
		Buccal cusps of one or more upper posterior teeth occlude on the buccal surface of the lower posterior teeth	Scissor bite
Spacing anomalies	Tooth displacement > 2 mm		Crowding
Dentition anomalies	Examination of all deciduous teeth		Agenesis
			Supernumerary teeth
			Dental malformations

The individual frequencies for each group of anomalies (occlusion, spacing and dentition) and for each type of anomaly (anterior crossbite, edge-to-edge, increased overjet, anterior open bite, deep overbite, posterior crossbite, scissor bite, crowding, agenesis, supernumerary teeth, or dental malformations) were calculated. The global prevalence of malocclusion was calculated considering the presence of at least one type of anomaly. Since more than one type of anomaly may be present in the same child, the sum of the types of frequencies may exceed the global prevalence of malocclusion.

The descriptive statistics of the variables included calculating the relative and absolute frequencies of the variables and, in the case of numerical variables, the mean and standard deviation. Inferential statistics, using the Chi-square test with a significance level of 5%, studied the relationship between malocclusion (global, by group and by type) and sex and age.

## Results

The final sample included 426 children with a mean age of 4.2 years (SD = 0.9). The characterization of the sample by sex, age, municipality, and type of kindergarten is shown in Table 2, as well as the respective comparison of this distribution with the one of the target-population. The participation rate was 92.2%. The reasons for the non-participation of 36 children were lack of authorization from the parents (*n* = 8), failure to return the consent form or questionnaire (*n *= 12), the child not being at school on the days of data collection (*n* = 13) or the child not assenting to the intraoral examination (*n* = 3).

**Table 2:** Distribution of the sample by sex, age, type of kindergarten and municipality, and respective comparison with the target population

	Study sample (*n* = 426)% (n)	Target population^a^ (*N* = 62,390)% (*N*)
Sex		
Male	53.5 (228)	51.5 (32,152)
Female	46.5 (198)	48.5 (30,238)
Age		
3 years	24.9 (106)	26.9 (16,805)
4 years	35.9 (153)	33.0 (20,596)
5 years	32.6 (139)	36.5 (22,760)
6 years	6.6 (28)	3.6 (2229)
Type of kindergarten		
PSSI	28.9 (123)	28.8 (17,974)
Private	31.9 (136)	30.9 (19,288)
Public	39.2 (167)	40.3 (25,128)
Municipality		
Amadora	7.5 (32)	6.5 (4051)
Cascais	10.3 (44)	9.9 (6160)
Lisboa	26.5 (113)	29.3 (18,284)
Loures	8.0 (34)	8.5 (5317)
Mafra	3.8 (16)	3.9 (2431)
Odivelas	3.8 (16)	5.4 (3400)
Oeiras	8.9 (38)	7.8 (4871)
Sintra	11.3 (48)	13.8 (8581)
Torres Vedras	3.3 (14)	3.3 (2069)
Vila Franca de Xira	8.5 (36)	6.4 (4015)
Grouped municipalities	8.2 (35)	5.2 (3211)

PSSI—Private Social Solidarity Institution

^a^ Data provided by the General Directorate of Education and Science Statistics (Ministry of Education, Science, and Innovation), 2015–2016

The global prevalence of malocclusion was 60.6% (*n* = 258). Considering the prevalence in each of the anomaly groups, occlusion anomalies had a prevalence of 59.2% (*n* = 252), spacing anomalies 1.6% (*n* = 7) and dentition anomalies 1.4% (*n* = 6) (Table 3). As shown in Table 4, within the group of occlusion anomalies, the most prevalent types were deep overbite (27%), increased overjet (22.1%) and posterior crossbite (14.1%). The least frequent types in this group were anterior crossbite (1.9%) and scissor bite (0.9%). In terms of spacing and dentition anomalies, there were 1.6% crowding, and 1.4% agenesis, respectively (Table 3).

**Table 3:** Malocclusion prevalence – total sample by sex and by age

	Global malocclusion		Occlusion anomalies		Spacing anomalies (Crowding)		Dentition anomalies (Agenesis)	
	% (*n*)	*p*	% (*n*)	*p*	% (*n*)	*p*	% (*n*)	*p*
Total (*n* = 426)	60.6 (258)		59.2 (252)		1.6 (7)		1.4 (6)	
Sex								
Male (*n* = 228)	57.5 (131)	0.16	55.3 (126)	0.08	1.8 (4)	0.85	1.3 (3)	0.86
Female (*n* = 198)	64.1 (127)		63.6 (126)		1.5 (3)		1.5 (3)	
Age								
3 years (*n* = 106)	69.8 (74)	0.08	69.8 (74)	**0.03**	0 (0)	0.26	1.9 (2)	0.88
4 years (*n* = 153)	58.2 (89)		56.9 (87)		2.6 (4)		1.3 (2)	
5/6 years *(n* = 167)	56.9 (95)		54.5 (91)		1.8 (3)		1.2 (2)	

*p* values in bold are statistically significant

**Table 4:** Distribution of the occlusion anomalies by sex and age

	Scissor bite		Anterior crossbite		Edge-to-edge		Open bite		Posterior crossbite		Increased overjet		Deep overbite	
	% (*n*)	*p*	% (*n*)	*p*	% (n)	*p*	% (*n*)	*p*	% (*n*)	*p*	% (*n*)	*p*	% (*n*)	*p*
Total (*n* = 426)	0.9 (4)		1.9 (8)		3.1 (13)		10.1 (43)		14.1 (60)		22.1 (94)		27.0 (115)	
Sex														
Male	1.3 (3)	0.39	1.8 (4)	0.84	3.1 (7)	0.98	10.5 (24)	0.75	11.8 (27)	0.15	18.0 (41)	**0.03**	28.5 (65)	0.45
Female	0.5 (1)		2.0 (4)		3.0 (6)		9.6 (19)		16.7 (33)		26.8 (53)		25.3 (50)	
Age														
3 years	1.9 (2)	0.27	0.9 (1)	0.68	2.8 (3)	0.35	14.2 (15)	0.11	16.0 (17)	0.79	30.2 (32)	0.06	30.2 (32)	0.17
4 years	0.0 (0)		2.0 (3)		4.6 (7)		11.1 (17)		13.7 (21)		20.9 (32)		21.6 (33)	
5/6 years	1.2 (2)		2.9 (4)		1.8 (3)		6.6 (11)		13.2 (22)		18.0 (30)		29.9 (50)	

*p* values in bold are statistically significant

Regarding the various parameters characterizing occlusion in the deciduous dentition (Table 5), the most common were bilateral class I canine relationship (57.3%), bilateral straight terminal plane (60.1%) and upper and lower type I Baume arch (53.3%). The majority of the sample had overjet and overbite values between 0 and 3 mm (76.2% and 63.1%, respectively). The overjet mean value was 2.6 mm (sd = 2.1), ranging from −1 to 11 mm, and the overbite mean value was 2.2 mm (sd = 2.1), ranging from −0.5 to 7 mm.

**Table 5:** Characterization of occlusal parameters in the deciduous dentition (*n* = 426)

A. Canine class				
		Right side		
		I	II	III
Left side	I	57.3% (*n* = 244)	10.1% (*n* = 43)	0.0% (*n* = 0)
	II	6.8% (*n* = 29)	24.2% (*n* = 103)	0.5% (*n* = 2)
	III	0% (*n* = 0)	0.2% (*n* = 1)	0.9% (*n* = 4)

B. Terminal plane of second primary molar				
		Right side		
		Mesial	Straight	Distal
Left side	Mesial	1.6% (*n* = 7)	0.5% (*n* = 2)	0.2% (*n* = 1)
	Straight	0.2% (*n* = 1)	60.1% (*n* = 256)	5.6% (*n* = 24)
	Distal	0% (*n* = 0)	4.2% (*n* = 18)	27.5% (*n* = 117)

C. Type of Baume arch			
		Upper arch	
		I	II
Lower arch	I	53.3% (*n* = 227)	5.2% (*n* = 22)
	II	1.9% (*n* = 8)	39.7% (*n* = 169)

D. Overjet				
Mean (sd)	Min.; Max	< 0 mm	0–3 mm	> 3 mm
2.6 (2.1)	−1; 11 mm	1.8% (*n* = 8)	76.2% (*n* = 324)	22.1% (*n* = 94)

E. Overbite				
Mean (sd)	Min.; Max	< 0 mm	0—3 mm	> 3 mm
2.2 (2.1)	−0.5; 7 mm	10.1% (*n* = 43)	63.1% (*n* = 269)	26.7% (*n* = 114)

There were statistically significant associations between age and the presence of occlusion anomalies (*p* = 0.03) (Table 3) and between sex and the presence of increased overjet (*p* = 0.03) (Table 4). In the first case, the prevalence of occlusion anomalies decreased with age, and, in the second case, the prevalence of increased overjet was higher in girls.

## Discussion

The main objective of this study was to determine the prevalence of malocclusion in a Portuguese preschool population. Epidemiological studies of malocclusion in the deciduous dentition, using representative samples, are scarce worldwide and in Portugal.

A general limitation of prevalence studies is the inability to obtain a representative sample. A random sample of the target population was obtained in the present study. However, not all the selected children were included in the study for different reasons stated above, despite the researchers’ best efforts, namely, returning several times to the same kindergartens to examine previously absent children and engaging the educators to motivate the parents to participate. These efforts resulted in a very high participation rate, allowing the results to be extrapolated to the children attending kindergartens in the Lisbon District (Portugal).

The global prevalence of malocclusion in the deciduous dentition (60.6%) may be considered very high. Prevalence values obtained in other studies with Portuguese populations provided range from 44% (Ventura 2005) to 78.8% (Furtado 2008). Gafaniz (2015) reported a prevalence of malocclusion in six-year-old children of 53%. More recently Ventura et al. (2021) found a global prevalence of malocclusion of 67.7% in children aged 2 to 6 years old. The prevalence of malocclusion in the deciduous dentition in other southern European countries ranged from 46.2% in Spain (Amr-Rey et al. 2022) to 50% in Italy (Grippaudo et al. 2020).

To what extent the variability of the prevalence values is explained by the use of different diagnostic criteria is unclear. But studies that used criteria comparable to those of the present study, found similar prevalence values. Such is the case, of two studies carried-out in Chinese populations, which obtained a prevalence of 66.3% (Zhou et al. 2016) and 68.3% (Lin et al. 2023), and one in an Indian population, with a prevalence of 69% (Singh et al. 2021),

The most prevalent type of malocclusion found in this study was deep overbite (27%), a result consistent with several other studies (Carvalho et al. 2011; Normando et al. 2015; Zhou et al. 2016, 2017; Shen et al. 2018). On the other hand, the least prevalent types of malocclusions were anterior crossbite (1.9%) and scissor bite (0.9%). These types of malocclusions are described as infrequent (Dimberg et al. 2015b; Bervian et al. 2016; Zhou et al. 2017). A systematic review referred a much higher prevalence of anterior crossbite (33.6%) (Shen et al. 2018). The prevalence of increased overjet was 22.1%, lower than other studies, which found higher prevalences of around 35% (Zhou et al. 2016, 2017; Zhang et al. 2017) and 42.7% (Ventura et al. 2021). In India, much lower prevalences were found, around 10% (Fernandes et al. 2017; Sharma et al. 2021).

Regarding the prevalence of space anomalies, crowding was found in only 1.6% of children, a result alike to those by Stahl and Grabowski (2003), Ventura (2005) and Fernandes et al. (2017). In China, Lin et al. (2023) found higher values for this type of malocclusion (10.5%).

The prevalence of dentition anomalies was low (1.4%), with only the presence of supernumerary teeth being found. This result is similar to other studies (Lochib et al. 2015; Sharma et al. 2021).

The most prevalent canine relationship was bilateral class I (64.1%), a common finding reported in the literature (Onyeaso and Sote 2002; Wagner and Heinrich-Weltzien 2015; Bervian et al. 2016; Zhang et al. 2017; Zhou et al. 2017; Sharma et al. 2021; Ventura 2021; Davidopoulo et al. 2022; Cabrera-Domínguez et al. 2023; Lin et al. 2023).

The most frequently observed terminal plane of the second molars was bilateral straight (64.8%), a result also found by other authors (Khan et al. 2014; Lochib et al. 2015; Zhang et al. 2017; Fernandes et al. 2017; Anu 2020; Sharma et al. 2021; Ventura et al. 2021; Cabrera-Domínguez et al. 2023; Lin et al. 2023). However, there are some studies that have found the mesial terminal plane to be the most frequent (Ventura 2005; Candido et al. 2010; Kumar and Gurunathan 2019).

The most frequent type of Baume arch was upper and lower type I, with generalized spaces and the existence of primate spaces. The same result was found by several other authors (Cândido et al. 2010; dos Santos et al. 2012; Ventura et al. 2021). This type of arch is more favourable to the positioning of permanent anterior teeth, with a lower risk of dental crowding.

This study found an association between the presence of occlusion anomalies and age (*p* = 0.03). It has been described that some types of malocclusions may worsen or improve with growth, while others remain unchanged. Bervian et al. (2016) found a reduction in open bite and increased overjet with increasing age and Singh et al. (2021), inversely, found a higher prevalence of malocclusion at the age of 3 years. The presence of malocclusion in the deciduous dentition poses an increased risk of developing malocclusion in the mixed and permanent dentitions (Shen et al. 2018). Overbite tends to worsen with growth (Dimberg et al. 2015b), with possible periodontal and functional problems, trauma, bruxism, clenching and temporomandibular joint dysfunction. In addition, some longitudinal studies show that increased overjet does not self-correct over time (Bishara et al.^1995^; Baccetti et al. 1997; Antonini et al. 2005). Posterior crossbite situations should be addressed early, as they can lead to functional mandibular deviation and, consequently, to an asymmetrical mandibular and/or craniofacial growth (Bell and Kiebach 2014; Bukhari et al. 2018).

There was also a statistically significant association between the presence of an increased overjet and sex (*p* = 0.03), with girls showing a higher prevalence. Singh et al. (2021) also found a higher prevalence of malocclusion in girls. On the other hand, Sepp et al. (2019) found a higher prevalence of increased overjet in boys. Nevertheless, most studies have not found a relationship between the presence of malocclusion and sex (Wagner and Heinrich-Weltzien 2015; Bervian et al. 2016; Zhou et al. 2016, Shen et al. 2018; Ventura et al. 2021; Amr-Rey et al. 2022; Rai et al. 2022; Lin et al. 2023).

The prevention of malocclusion in the deciduous dentition is fundamental and involves, above all, the early adoption of favourable behaviours to avoid incorrect craniofacial development and tooth positioning. These behaviours include breastfeeding, reducing or eliminating non-nutritive sucking habits, nasal breathing, alternating unilateral chewing, consumption of hard and fibrous foods, and good oral hygiene and fluoride exposure to prevent caries.

The adoption of the above-mentioned behaviours rests essentially on education of the parents and caregivers. Oral health promotion should begin when the mother is pregnant and in the first years of the child’s life, therefore all health professionals involved (obstetricians, family doctors, paediatricians, dentists and dental hygienists) have an important responsibility in this process. Additionally, it is important to evaluate the child's occlusion at an early age, not only to correct harmful behaviours, but also, if needed, to refer the child for early orthodontic treatment.

## Conclusions

Considering the results obtained in this study, it can be concluded that:

- The prevalence of malocclusion in the deciduous dentition in the population of the Lisbon District was high.
- Deep overbite and increased overjet were the most prevalent types of malocclusions.
- Class I canine, straight terminal plane of the second deciduous molars and type I Baume arch were the most frequently found characteristics of the deciduous dentition.
- There was a statistically significant association between age and the presence of occlusion anomalies, with their prevalence decreasing with increasing age, and between sex and the presence of increased overjet, with a higher prevalence in girls.

## Data Availability

Authors declare data transparency and if necessary, availability of data and material.
